# Propagation and self-healing properties of Bessel-Gaussian beam carrying orbital angular momentum in an underwater environment

**DOI:** 10.1038/s41598-018-38409-2

**Published:** 2019-02-14

**Authors:** Shengmei Zhao, Wenhao Zhang, Le Wang, Wei Li, Longyan Gong, Weiwen Cheng, Hanwu Chen, Jozef Gruska

**Affiliations:** 1 Institute of Signal Processing and Transmission, Nanjing University of Posts and Telecommunications(NUPT), Nanjing, 210003 China; 2 Information Physics Research Center and Department of Applied Physics, NUPT, Nanjing, China; 3 School of computer science and engineering, Southeast University, Nangjing, China; 4 Faculty of informatics, Masaryk University, Botanická 68a, Brno, 60200 Czech Republic

## Abstract

In this paper, we report on experimental demonstration of the propagation and self-healing property of Bessel-Gaussian (BG) beam carrying orbital angular momentum (OAM) in an underwater environment. Especially, the effects of topological charge, temperature gradient, and salinity on the transmission and self-reconstruction of BG beam in underwater turbulence are analyzed. The results show that the detection probabilities both for propagation and self-healing greatly decrease with temperature gradient, and gradually decrease with salinity. BG beam has a self-healing property in the underwater environment when the obstruction is quite small. The detection probability greatly decreases with obstruction size, while it gradually decreases with salinity fluctuations for different obstruction sizes. For the same blockage ratio, the smaller topological charge of BG beam is, the better self-healing characteristics the BG beam has.

## Introduction

Underwater wireless optical communication (UWOC) is expected to provide high data rates to transmit large data over several tens of meters by using a suitable wavelength (in the blue/green range)^1^.

With a huge increase of underwater applications, such as divers, unmanned underwater vehicles, submarines, and sensors in the oceanic environment^2,3^, UWOC has currently received more and more attention.

Recently published results concerning UWOC have demonstrated that the usage of polarization and wavelength modulation could increase the data rate significantly^4^. Recent reports have also explored the feasibility of using orbital angular momentum (OAM), a promising degree of freedom for the fundamental studies in the quantum and free-space optical communications^5–12^, to spatially multiplex simultaneous data streams in underwater environments^13–15^.

It is already well-known that the optical turbulence in underwater environments, such as in oceanic environments, is mainly induced by temperature and salinity fluctuations. This turbulence has severely degraded the communication quality of the OAM-based UWOC system^16–19^. For example, in^16^ there is a theoretical study of the influence of temperature and salinity fluctuations on the average intensity of Gaussian Schell-model vortex beams in the turbulent ocean that showed that partially coherent beams have more robust turbulence resistance than fully coherent ones. The propagation of partially coherent Laguerre-Gaussian beams in the turbulent ocean was also discussed in^17^. In addition, the effect of the ocean turbulence on the channel capacity of an oceanic OAM-based UWOC systems was studied in^18^. The statistical properties, such as the average intensity, polarization, and coherence of the vortex beams propagation in oceanic turbulence, have also been theoretically investigated in^16,19^.

Non-diffraction vortex beams, carrying OAM with helical phase fronts, were widely adopted as beam sources to mitigate the effect of the turbulence on any optical vortex^20,21^. A Bessel-Gaussian (BG) beam is an important member of the family of pseudo-nondiffraction vortex beams. The features of the Bessel beam provide a new dimension to code/decode data information on OAM state, and the theoretical infinity of the topological charge enables possible high-dimensional structured light coding/decoding for optical communications^22,23^.

In addition, BG beams have the ability to recover itself in the case of some obstructions, which is important for optical communications that rely on the line-of-sight operations^24^. Therefore, the non-diffraction and self-healing characteristics of BG beams enable them to be a promising resource for the OAM-based UWOC systems. Recently, the propagation property of BG beams in the optical free space was discussed in^25^, and the obstruction-free property of BG beams in a free-space optical communication system was analyzed in^22^. However, the special properties of BG beams in underwater environments have not been explored yet.

In this paper, we will present experimental analysis of the propagation and self-healing properties of BG beams in underwater environments. We used a one-meter rectangular tank with the distilled water to simulate the underwater environment. The temperature fluctuations was controlled by a heater inside the tank, and salinity fluctuations was obtained by dissolving different weights of salt in the water. Simultaneously, an opaque bar with different sizes was placed before the tank and along the propagation path to simulate obstructions. The impact of the temperature gradient and salinity on the propagation and self-healing property was explored, and the power loss for the propagation and self-healing procedure was discussed.

## Results

### Theoretical analysis

The light field of BG beams carrying OAM is expressed as

1 $${{\rm{\Phi }}}_{l}(r,\theta )=C\,\exp (\frac{-{r}^{2}}{{w}_{0}^{2}}){J}_{l}(\frac{2\pi r}{{r}_{0}})\exp (jl\theta )$$

where *exp*(*jlθ*) is the spatial phase of the field, which causes vortex wavefront. *r* and *θ* are the polar coordinates at the transmitted plane. *J*_*l*_(⋅) is the *l*th-order Bessel function. *r*_0_ is a parameter related to the BG beams radius. *w*_0_ is the zero-order Gaussian radius at the waist. *l* represents the OAM topological charge. *C* is a normalization factor.

Due to BG beams are susceptible to the oceanic turbulence and obstacle during the propagation, the received light field Φ′_*l*_(*ρ*, φ) can be expressed using the extended Huygens-Fresnel principle^26^ as

2 $${{\rm{\Phi }}}_{l}^{^{\prime} }(\rho ,\phi )=\int \alpha {{\rm{\Phi }}}_{l}(r,\theta )h(r,\theta ,\rho ,\phi )O(r,\theta )r{\bf{d}}r{\bf{d}}\theta $$

where *ρ* and *φ* are the polar coordinates at the received plane, *α* is the attenuation coefficient due to absorption and scattering, and *O*(*r*, *θ*) represents the obstacle. *h*(*r*, *θ*, *ρ*, *φ*) denotes the paraxial Green’s function for oceanic propagation^26^,

3 $$h(r,\theta ,\rho ,\phi )=\frac{\exp (jkL+jk\frac{{r}^{2}+{\rho }^{2}-2r\rho \,\cos (\theta -\phi )}{2L})}{j\lambda L}{\rm{e}}{\rm{x}}{\rm{p}}(\chi (r,\theta ,\rho ,\phi )+j\varphi (r,\theta ,\rho ,\phi ))$$

where *λ* is the wavelength, *L* is the length of the path and *k* = 2*π*/*λ* is the wave number. *χ*(*r*, *θ*, *ρ*, *φ*) and *ϕ*(*r*, *θ*, *ρ*, *φ*) describe the stochastic log-amplitude and phase fluctuations caused by oceanic turbulence, respectively.

Because BG beams are affected by oceanic turbulence and obstacle, the helical wavefronts of BG beams are damaged and thus the power of a single OAM mode of BG beams is spread to other OAM modes of BG beams, which causes the crosstalk between different OAM modes of BG beams. Hence, the received light field ${\Phi }_{l}^{^{\prime} }(\rho ,\phi )$ can be expressed as a superposition of various BG beams with different OAM modes ${{\rm{\Phi }}}_{m}(\rho ,\phi )=$ $C\,\exp (\,-\,{\rho }^{2}/{w}_{0}^{2}){J}_{m}(2\pi \rho /{r}_{0})\exp (jm\phi )$,

4 $${{\rm{\Phi }}}_{l}^{^{\prime} }(\rho ,\phi )=\sum _{m=-\infty }^{+\infty }{\gamma }_{m}{{\rm{\Phi }}}_{m}(\rho ,\phi )$$

where *γ*_*m*_ is the superposition coefficient of OAM modes *m*, which can be given by

5 $${\gamma }_{m}=\int {{\rm{\Phi }}}_{m}^{\ast }(\rho ,\phi ){{\rm{\Phi }}}_{l}^{^{\prime} }(\rho ,\phi )\rho {\bf{d}}\rho {\bf{d}}\phi $$

where * is the complex conjugate. The probability remaining in transmitted OAM mode *l*, named detection probability, is

6 $${P}_{l}=\frac{|{\gamma }_{l}{{\rm{\Phi }}}_{l}(\rho ,\phi {)|}^{2}}{|{{\rm{\Phi }}}_{l}^{^{\prime} }(\rho ,\phi {)|}^{2}}=\frac{|{\gamma }_{l}{|}^{2}}{\sum _{m=-\infty }^{+\infty }|{\gamma }_{m}{|}^{2}}$$

and the crosstalk probability from the transmitted OAM mode *l* to the received OAM mode *l*′ is

7 $${P}_{l\to l^{\prime} }=\frac{|{\gamma }_{l^{\prime} }{{\rm{\Phi }}}_{l^{\prime} }(\rho ,\phi {)|}^{2}}{|{{\rm{\Phi }}}_{l}^{^{\prime} }(\rho ,\phi {)|}^{2}}=\frac{|{\gamma }_{l^{\prime} }{|}^{2}}{\sum _{m=-\infty }^{+\infty }|{\gamma }_{m}{|}^{2}}$$

### Experimental setup

**Figure 1:**
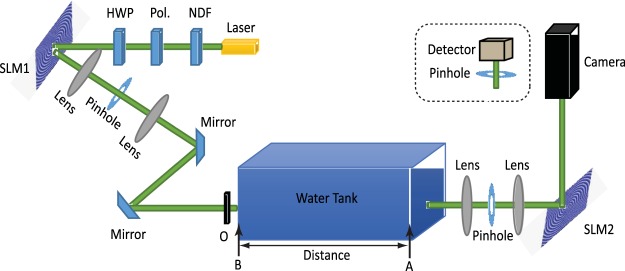
The experimental setup for analyzing BG beam properties in underwater environments: NDF - neutral density filter; Pol. - polarizer; HWP - half-wave plate; SLM - spatial light modulator; O - obstruction object.

Here, we mainly analyze the propagation and self-healing properties of BG beam carrying OAM by experimental method. Figure 1 depicts the experimental setup for an analysis of BG beam properties in underwater environments. A Gaussian beam with wavelength 532 *nm* is emitted from a laser diode (Thorlabs, CPS532), and then a neutral density filter (NDF) is used to attenuate the intensity of the Gaussian beam. After passing through the polarizer (Pol.) and half-wave plate (HWP), the Gaussian beam matches its polarization to the optimized working polarization of the selected polarization-sensitive spatial light modulator (SLM). When the polarized Gaussian beam is illuminated on a SLM (SLM1, Holoeye, PLUTO-VIS-006-A), the desired BG mode is generated, where the special phase hologram grating is displayed on the liquid crystal screen of the SLM. Here, the special hologram grating is

8 $$H(x,y)=\frac{1}{2}\{1+\,\cos [2\pi \frac{\sqrt{{x}^{2}+{y}^{2}}}{{r}_{0}}+l\,\arctan (\frac{y}{x})-2\pi Kx]\},$$

where *l* is the topological charge of BG beam desired to produce, *r*_0_ is a parameter related to the BG beams radius, and *K* is is the number of grating lines per unit length. A spatial filter consisting of two lenses and a pinhole is used to produce a clean BG beam.

The BG beam carrying OAM mode is then transmitted through a water tank simulating the underwater environment. The propagation distance is 1 *m*. An opaque bar located in the beam path represents an obstruction. The obstruction is placed before the water tank and the size of the bar is controllable. A heater is placed at one side of the tank (Point A)^27^, and the temperature gradient is calculated by the temperature difference of point B and A over the distance between the two points. Salt is used to produce different salinity. The salinity is produced by different weights of salt dissolved in water, whose value is calculated by the weight of salt over the weight of water. As a result, part of the energy launched into a single OAM mode is redistributed into other OAM modes after the underwater turbulent propagation. After passing through a spatial filter, the BG beam illuminates another SLM (SLM2, Holoeye, PLUTO-VIS-006-A).

Note that there are four reflections, two mirrors reflections and two SLM reflections, in the experimental setup. Each reflection will change the sign of the topological charge, that is, the topological charge with $\ell$ of a BG beam changes to $-\ell$ after a reflection, except $\ell =0$. Hence, the BG beam turns back to Gaussian mode when the spiral phase pattern loaded on SLM2 is the same as that on SLM1. The resultant beam has a bright center, while the centers of others OAM modes are still dark due to phase singularities. Finally, a camera (Thorlabs, BC106N-VIS/M) is used to detect the intensity profile of the output beam, and which is replaced by a pin hole with a power sensor (Thorlabs S120) when energy *E*_*l*_ is needed to detect. Here, we use a pin hole with a power sensor to obtain the energy of the desired OAM mode, which was also used in^14^.

In the experiment, we first used the same hologram grating on SLM2 as that on SLM1 (for example, the phase pattern for $\ell$ topological charge OAM mode) to obtain the energy (${E}_{\ell }$) of the desired receiving OAM mode by Thorlabs S120, where the pin hole (before the power sensor) was utilized to only past the Gaussian mode. The size of the pin hole was adjusted to pass all the Gaussian mode energy. Then, we kept the pin hole and the power sensor no change, and replaced another hologram gratings displayed onto SLM2 (corresponding to other OAM modes’ phase pattern) to detect other receiving OAM modes energy (${E}_{m},m\ne \ell$). The operation was repeated until all the energies of the possibly receiving OAM modes had been detected. The detection probability ${P}_{\ell }$, denotes the ratio of energy of the desired OAM mode over all possibly received OAM mode energy, changes to,

9 $${P}_{l}=\frac{{E}_{\ell }}{\sum _{m,m\ne \ell }{E}_{m}+{E}_{\ell }},$$

where ${E}_{\ell }$ denotes the energy for the desired receiving OAM mode, and ${E}_{m},m\ne \ell$ represents other possibly receiving OAM modes’s energy.

### Experimental results and analysis

**Figure 2:**
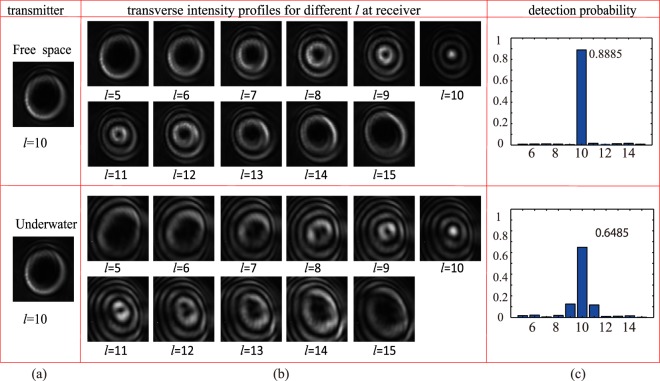
The effect of underwater optical turbulence on the propagation property of BG beam, together with results in a free space environment.

We first demonstrate, see Fig. 2, the effect of underwater environments on the propagation properties of BG beam, in comparison with the results in a free space propagation. Figure 2(a) shows the transverse intensity profile of $\ell =10$ BG beam at transmitter. Figure 2(b) shows the transverse intensity profiles for different $\ell$ BG beams (from $\ell =5$ to $\ell =15$) at receiver, Fig. 2(c) shows the detection probability of $\ell =10$ BG beam also at the receiver side. The temperature was 23.60 °C, the temperature gradient was 0.10 °*C*/*m*, and the salinity was 0. Different spiral phase patterns were used on SLM2 to demodulate the BG beam at the receiver side, such as $\ell =5$ to $\ell =15$. The results received showed that the BG beam with $\ell =10$ was demodulated with the corresponding phase hologram grating. In particular, it emerged a bright spot at the center of the received beam. On the other side, the other received beams with $\ell$ from 5 to 15, except 10, still had dark centers due to the phase singularity. Comparing with the results in the free space environment, the transverse intensity profiles in the underwater environment had more noise. This indicated that the underwater turbulence induced more serious OAM mode crosstalk and spiral spectrum spread. Moreover, the detection probability of $\ell =10$ at the receiver side further demonstrated that underwater turbulence had a more severe interference on the propagation property. The detection probability was 0.6485 for the underwater turbulence, while it was 0.8875 for the atmospheric optical turbulence with the same topological charge, temperature, and propagation distance. At the same time, the underwater turbulence distributed more energy to the adjacent modes.

**Figure 3:**
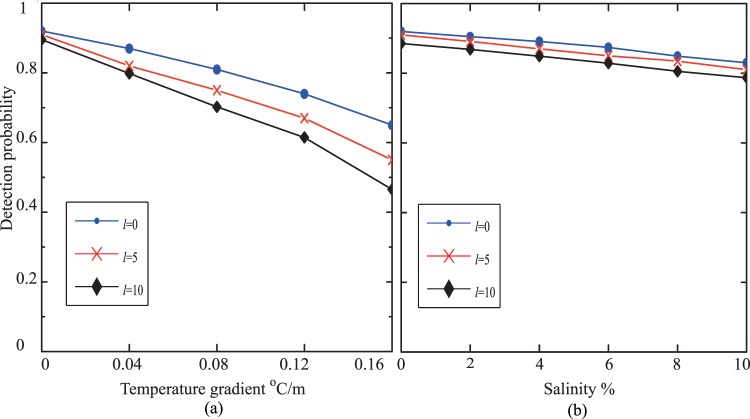
The propagation property of BG beam in an underwater environment. (**a**) The detection probability against temperature gradient (**b**) the detection probability against salinity.

Since the optical turbulence in an underwater environment may be induced by temperature fluctuations and salinity fluctuations, we have explored and demonstrated the underwater propagation properties of BG beams against temperature gradient in Fig. 3(a) and against salinity in Fig. 3(b). The temperature was 23.56 °C. BG beams with different topological charges $\ell (\ell =\mathrm{0,}\,\mathrm{5,}\,\mathrm{10)}$ were considered. The results showed that the propagation properties of BG beams were much influenced by temperature gradients, but were little effected by salinity. The detection probabilities decreased significantly with the increase of temperature gradients. For $\ell =10$, the detection probability was 0.895 when the temperature gradient was 0 °*C*/*m*, while it dropped to 0.465 when the temperature gradient was up to 0.16 °*C*/*m*. They gradually decreased from 0.912 to 0.813 when the salinity varied from 2% to 10% for $\ell =5$. The results received also indicated that a larger topological charge BG beam had a worse propagation property against temperature gradient and salinity. BG beams with larger topological charges had a big worse interference with the temperature fluctuations, but only a bit worse interference with the salinity fluctuations.

**Figure 4:**
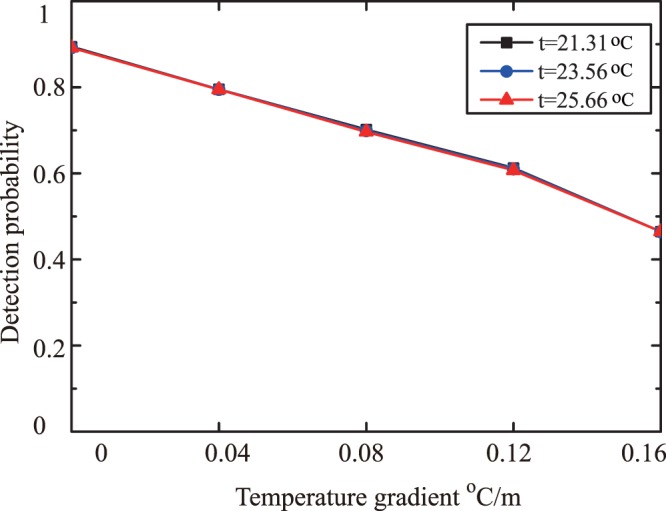
The detection probability against temperature gradient with different temperature in underwater environment.

In order to study the relation of the detection probability of BG beam in an underwater environment to temperature and temperature gradient, we present the detection probability versus temperature gradient under different temperature in Fig. 4. The temperature was setup to 21.31 °*C*, 23.56 °*C*, 25.66 °*C*, respectively. The temperature gradient varied from 0 °*C*/*m* to 0.16 °*C*/*m*. The BG beam with topological charges $\ell =10$ was used. The results showed that the detection probability varied quickly with temperature gradient, however, there was a little influence of temperature on the detection probability for different temperature gradient.

**Figure 5:**
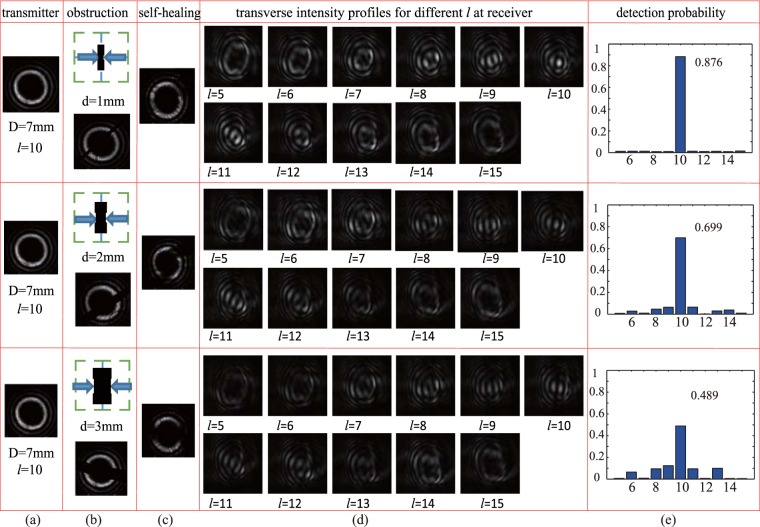
The self-healing properties of BG beam in underwater environments.

We have investigated and will now report also the self-healing property of BG beams in underwater environments. The obstruction considered was an opaque vertical bar with width *d*, located at the center of the beam’s propagation and before the tank, where *d* was set to 1 *mm*, 2 *mm*, 3 *mm* in the experiment. Figure 5 shows the self-healing property of BG beam in an underwater environment. (Here, the diameter of the BG beam was 7*mm*, the topological charge $\ell$ of the BG beam was 10). The BG beam with $\ell =10$ was produced and the diameter of its intensity profile was made to be 7 *mm* by a lens. Figure 5(a) shows the transverse intensity profile for $\ell =10$ BG beam at transmitter, Fig. 5(b) shows the obstruction and the transverse intensity after the obstruction, Fig. 5(c) shows the self-healing property of BG beam after a propagation, Fig. 5(d) shows the transverse intensity profiles for different $\ell$ (from $\ell =5$ to $\ell =15$) at receiver, and Fig. 5(e) shows the detection probability of $\ell =10$ at the receiver side. The temperature was 22.97 °*C*, there were no temperature gradient and salinity. We could clearly see that BG beam was reconstructed after 1*m* underwater propagation when the obstruction is 1*mm*, quite small. The gap caused by the obstruction had been made up after the propagation. Also, there was a bright spot at the center of the transverse intensity profile of $\ell =10$ when *d* was 1 *mm*, which indicated that BG beams had self-healing property. As the obstruction size increasing, the center spot became more vague, hindering the self-healing ability of BG beam. We could obtain the same result from the detection probability at the receiver side. In order to quantify the extent of blockage, we define the blockage ratio as the width of the obstruction over the diameter of the inner ring of BG beam. It was shown that the probability of detecting $\ell =10$ mode at the receiver was 0.876 when the blockage ratio was 14.29%, and the value dropped to 0.699 when the blockage ratio was 28.57%, and the value was 0.489 when the blockage ratio was 42.86%. It was also shown that there was more mode crosstalk when *d* was 3 *mm*.

**Figure 6:**
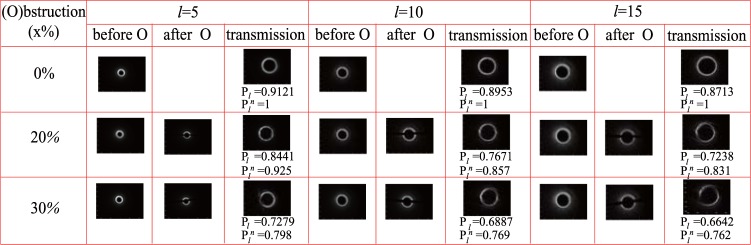
The self-healing characteristics subjected to the obstruction in an underwater environment. O represents obstruction. *P*_*l*_ is the detection probability, and ${P}_{l}^{n}$ is the normalized detection probability.

We further demonstrate the self-healing characteristics subjected to the obstruction in an underwater environment in Fig. 6. The temperature was 25.9 °*C*, and there were no temperature gradient and salinity. Different topological charges $\ell =5,\,10,\,15$ BG beams were demonstrated with different blockage ratios (0%, 20%, 30%). Here, the normalized detection probability ${P}_{l}^{n}$ is defined as

10 $${P}_{l}^{n}=\frac{{P}_{l}(x)}{{P}_{l}\mathrm{(0)}},$$

where *P*_*l*_(*x*) is the detection probability with *x*% blockage ratio, and *P*_*l*_(0) denotes the detection probability without obstruction. The results showed that BG beams could be repaired after the obstructed beams propagating a distance (1 *m*). BG beam had an excellent self-healing property in the underwater environment. The self-healing property reduced with the blockage ratio increase. For the same blockage ratio, the smaller topological charge of BG beam was, the better self-healing characteristics the BG beam had. For 20% blockage ratio, the normalized detection probability of BG beam with $\ell =5$ still was 0.925, while they were reduced to 0.857 and 0.831, respectively, for $\ell =10$ and $\ell =15$. However, they were almost the same when the blockage ratio was equal to 30%.

**Figure 7:**
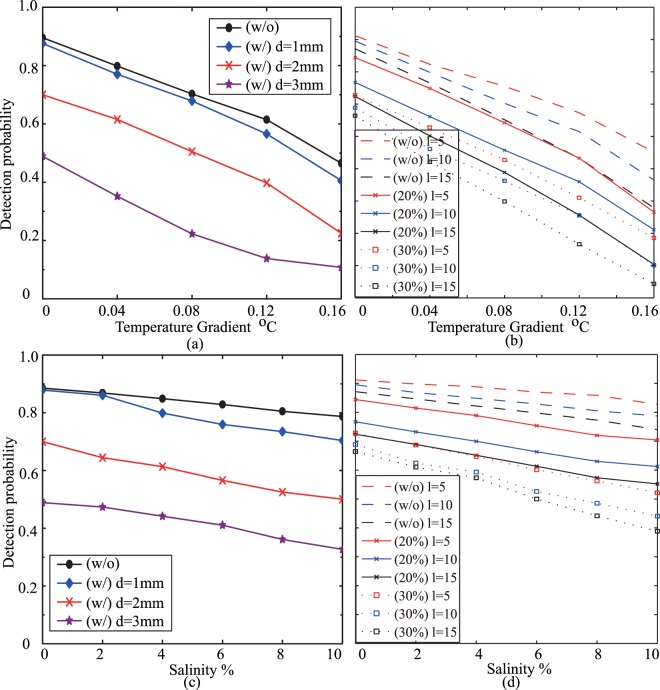
The self-healing property of BG beams against temperature and salinity in an underwater environment.

Finally, we discuss the temperature fluctuations and salinity fluctuations on the self-healing property of BG beams in underwater environments in Fig. 7, where Fig. 7(a) shows detection probability of $\ell =10$ against temperature gradient for different size obstructions (*d* = 1 *mm*, 2 *mm*, 3 *mm*), Fig. 7(b) shows detection probability for different topological charges $\ell$ ($\ell =\mathrm{5,10,15}$) when the blockage ratio is 0%, 20%, 30%, respectively, Fig. 7(c) shows detection probability of $\ell \mathrm{=10}$ against salinity for different size obstructions, while Fig. 7(d) shows detection probability for different topological charges $\ell$ against salinity. Still the blockage ratio is selected as 0%, 20%, and 30%. The temperature was 25.9 °*C*. The results showed that the BG beam had a good self-healing property when the size of the obstruction was small, say, less than 1/7 ≈ 14%. With an increase of obstruction size, the detection probability decreased significantly with the temperature gradient increase. The detection probability was 0.895 for no obstruction, was 0.876 for *d* = 1 *mm*, was 0.70 for *d* = 2 *mm*, and was 0.489 for *d* = 3 *mm* when the temperature gradient was 0 °*C*/*m*, while they were 0.465, 0.405, 0.225 and 0.108, respectively, when the temperature gradient was 0.16 °*C*/*m*. The results also showed the detection probability decreased with the topological charge when the same blockage ratio was used for the different topological charge BG beams. A larger topological charge BG beam had a worse detection probability after the underwater turbulence for the same blockage ratio. On the other hand, the detection probability greatly decreased with obstruction size, while it gradually decreased with salinity fluctuations for different obstruction sizes.

## Discussion

In this paper, we have presented results of our experimental demonstration of the propagation property and the self-healing property of BG beam carrying OAM mode in an underwater environment. The effects of the OAM topological charge, temperature gradient and salinity on the propagation and self-healing of BG beam in the underwater turbulence environment were analyzed. The results have shown that the underwater optical turbulence has a more severe interference effect on the propagation property of BG beams, the detection probabilities in the propagation procedure greatly decrease with temperature gradient, and gradually decrease with salinity. Moreover, a more severe interference is caused for a larger topological charge BG beam with the same underwater turbulent environment. On the other hand, BG beam may still have self-healing characteristics in an underwater environment when the obstruction is quite small. The detection probability in self-healing procedure greatly decrease with temperature gradient, and gradually decrease with salinity. The self-healing property is significantly affected by the size of the obstruction.
